# Nutrition, Gut Microbiota, and Epigenetics in the Modulation of Immune Response and Metabolic Health

**Published:** 2025-05-05

**Authors:** Sabrin Bacaloni, Devendra K Agrawal

**Affiliations:** Department of Translational Research, College of Osteopathic Medicine of the Pacific, Western University of Health Sciences, Pomona CA 91766, USA

**Keywords:** Dietary patterns, DNA methylation, Epigenetics, Gut microbiota, Histone modification, Immune dysfunction, Immune regulation, Immunity, Inflammation, Macronutrients, Mediterranean diet, Micronutrients, MicroRNAs, Nutrition, Plant-based diet, Short-chain fatty acids, Obesity, Type 2 diabetes

## Abstract

Immune system function is intricately shaped by nutritional status, dietary patterns, and gut microbiota composition. Micronutrients such as vitamins A, C, D, E, B-complex, zinc, selenium, iron, and magnesium are critical for maintaining physical barriers, supporting immune cell proliferation, and regulating inflammation. Macronutrients—including proteins, fats, and carbohydrates—also modulate immune responses through their impact on immune metabolism and the gut-immune axis. Epigenetic mechanisms, including DNA methylation, histone modifications, and microRNA expression, mediate the long-term effects of diet on immune function and tolerance. Diet-induced alterations in gut microbiota further influence immune homeostasis via microbial metabolites like short-chain fatty acids. Imbalanced diets, particularly the Western diet, contribute to immune dysregulation, chronic inflammation, and the development of metabolic disorders such as obesity and type 2 diabetes. While plant-based and Mediterranean dietary patterns have shown anti-inflammatory and immunoregulatory benefits, gaps remain in understanding the long-term epigenetic impacts of these diets. This review integrates current knowledge on how nutrition and the microbiome regulate immunity, highlighting future directions for personalized dietary strategies in preventing chronic immune-related conditions.

## 1. Introduction

The immune system is a complex network of tissues, organs, cells, and signaling molecules that protect the host from infections and harmful substances. It consists of two interconnected systems: innate immunity, present from birth, which provides the first line of defense through physical and chemical barriers such as the skin, mucus secretions, and stomach acidity, and adaptive immunity, which develops over time and provides a specific response by recognizing and remembering pathogens [1,2].

Individual variations in immune function are influenced by factors such as genetics, age, sex, smoking, physical activity, alcohol intake, diet, menstrual cycle phase, and stress [3]. Among these factors, nutrition plays a fundamental role in modulating immune responses, influencing immune cell development and inflammation regulation [4].

Both micronutrients, such as vitamins and minerals, and macronutrients, including carbohydrates, lipids, and proteins, contribute to immune function, while dietary patterns can either support immune resilience or promote dysfunction [4]. Additionally, epigenetic modifications such as DNA methylation and histone modifications mediate the long-term effects of diet on immune regulation [5–7].

The gut microbiota serves as a crucial link between diet and immunity, regulating immune homeostasis, inflammation, and metabolic health [8–10]. Certain dietary patterns can enhance microbiota diversity and promote beneficial metabolites such as short-chain fatty acids (SCFAs), while others may drive dysbiosis and inflammation [8,11–13].

Given the rising prevalence of metabolic disorders like obesity and diabetes—which are closely linked to chronic inflammation and immune dysfunction—understanding the interplay between diet, gut microbiota, immunity, and metabolism is critical [14–21].

This review explores how nutrition influences immune responses, the role of gut microbiota and epigenetic factors in this relationship, and the implications for metabolic conditions such as obesity and diabetes.

## 2. Micronutrients and Immunity

**Figure 1:**
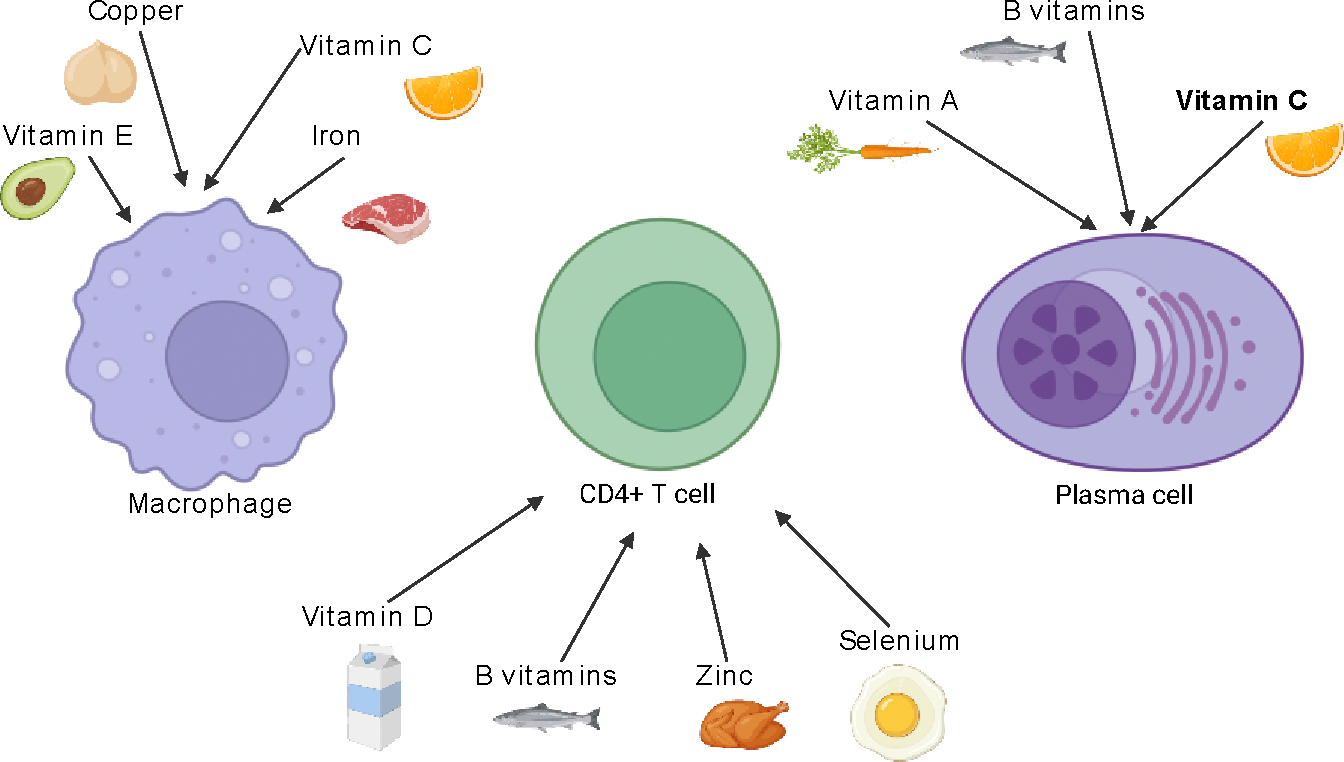
Micronutrients and Their Roles in Immune Cell Function: This diagram illustrates the interaction of key micronutrients with major immune cells: macrophages, CD4+ T cells, and plasma cells. Vitamins A, C, D, E, B-complex, zinc, selenium, iron, and copper each influence immune cell development, cytokine signaling, oxidative stress regulation, and antibody production. These nutrients modulate both innate and adaptive immunity through diverse mechanisms including epithelial barrier support, free radical scavenging, and regulation of immune gene expression.

Micronutrients, including essential vitamins and trace minerals, play indispensable roles in regulating immune function. They support the structural integrity of physical barriers, aid in the development and function of immune cells, and modulate inflammatory and oxidative stress pathways [1–3]. Deficiencies in these nutrients can compromise immunity, leading to increased susceptibility to infections, chronic inflammation, and autoimmunity (Figure 1).

### 2.1 Vitamins Supporting Immune Function

#### 2.1.1 Vitamin A (Retinoic Acid) in Mucosal Immunity and Immune Cell Regulation:

Vitamin A is vital for maintaining mucosal barriers and modulating adaptive immune responses. Its active metabolite, retinoic acid (RA), promotes gut-specific immune responses by inducing the expression of gut-homing markers, such as α4β7 integrin and CCR9 on T cells, directing them to the intestinal mucosa [22]. RA interacts with retinoic acid receptors (RARs) in T cells to regulate transcriptional programs that guide lymphocyte migration and differentiation, helping establish mucosal immune surveillance. Additionally, RA enhances B-cell immunoglobulin production, particularly secretory IgA, which is crucial for defending mucosal surfaces against pathogens [23]. Secretory IgA binds and neutralizes pathogens and toxins in the gut lumen, preventing their translocation across the epithelium and limiting immune activation. Through these mechanisms, vitamin A strengthens both innate and adaptive immunity at critical barrier sites. Its dual role makes it an integral component of mucosal immunity, contributing to both immune tolerance and effective pathogen defense (Figure 1).

#### 2.1.2 Vitamin C in Antioxidant Defense and Immune Cell Support:

Vitamin C is a potent antioxidant that protects immune cells from oxidative damage during inflammatory responses. As a water-soluble vitamin, it directly scavenges reactive oxygen species (ROS) and regenerates other antioxidants like vitamin E, helping to control oxidative stress at sites of infection. It reinforces epithelial barrier integrity, enhances the function of phagocytes, and supports the proliferation and function of T and B lymphocytes [24]. Specifically, it promotes neutrophil migration to infection sites and enhances their ability to engulf and kill pathogens through oxidative bursts, while also facilitating apoptosis and clearance of spent neutrophils to resolve inflammation. A deficiency in vitamin C impairs multiple aspects of immune defense and is associated with increased susceptibility to respiratory and other infections. This includes reduced interferon production and impaired lymphocyte function, making the body more vulnerable to viral and bacterial invasion, particularly in the lungs and mucosal tissues.

#### 2.1.3 Vitamin D-induced Immunomodulation and Autoimmune Regulation:

Vitamin D is well-recognized for its immunomodulatory properties. It enhances innate immune defenses by promoting the production of antimicrobial peptides like cathelicidins, which help neutralize pathogens and strengthen the first line of defense at epithelial surfaces [25–34]. In addition to its role in innate immunity, vitamin D critically influences adaptive immune responses. It promotes the differentiation and function of regulatory T cells (Tregs), which help maintain immune tolerance and prevent overactive immune responses that could lead to tissue damage or autoimmunity. This regulatory effect also involves the suppression of pro-inflammatory cytokines and a shift away from Th1 and Th17 responses, both of which are implicated in autoimmune diseases [35–37]. In addition, vitamin D is a potent immunomodulator playing a significant role in preventing insulin resistance, macrophage polarization, cholesterol efflux, cardiac hypertrophy, inflammation in occlusive vascular diseases, esophageal diseases, and inflammatory bowel disease [38–44].

Clinical trials demonstrate that vitamin D supplementation improves outcomes in severe COVID-19 cases, potentially by attenuating cytokine storms and hyperinflammation, mechanisms that are often driven by uncontrolled immune activation [35,45]. Moreover, in aging populations where immunosenescence reduces immune responsiveness and increases inflammation, vitamin D has been shown to help preserve immune function. It not only supports T cell function but also reduces chronic inflammation markers, which are commonly elevated in older adults [46]. These combined effects underscore the importance of maintaining adequate vitamin D levels for both protective immunity and long-term immune regulation (Figure 1).

#### 2.1.4 Vitamins B6, B12, and Folates (B9) regulating DNA Synthesis and Lymphocyte Function:

B vitamins are essential for cellular proliferation and immune cell function, particularly because of their roles in one-carbon metabolism and nucleic acid synthesis. Vitamin B6 plays a multifaceted role in immune regulation by supporting the differentiation of lymphocytes and modulating cytokine production. It is involved in the synthesis of several neurotransmitters and amino acids that influence lymphocyte communication and maturation.

Vitamins B12 and folate are especially critical for DNA synthesis, methylation, and repair in rapidly dividing immune cells. These processes are indispensable for the clonal expansion of T and B lymphocytes during immune responses. Folate also contributes to the synthesis of purines and thymidylate, building blocks of DNA, while B12 is a cofactor in the conversion of homocysteine to methionine, which is required for methylation reactions. Deficiencies in any of these B vitamins can impair both humoral and cell-mediated immunity, reducing antibody production and weakening T cell responses, thereby increasing susceptibility to infections [33].

Beyond immune defense, these vitamins may influence epigenetic programming in immune cells, particularly through DNA methylation, potentially affecting long-term immune function and tolerance. This suggests that maintaining adequate B vitamin status is important not only for immediate immune responses but also for long-term immune regulation.

### 2.2 Essential Trace Elements in Immune Regulation

#### 2.2.1 Zinc in T Cell Development and Antioxidant Support:

Zinc is essential for immune function, particularly for the development and activation of T lymphocytes. It supports thymic hormone activity, stabilizes cell membranes, and regulates gene expression through zinc-finger transcription factors. Zinc also acts as an antioxidant, protecting immune cells from oxidative stress during inflammation. Deficiency impairs both innate and adaptive immunity, leading to reduced T cell numbers, compromised barrier function, and increased infection risk. Even marginal deficiencies can significantly weaken immune defenses, especially in vulnerable populations [1,2].

#### 2.2.2 Selenium in Antioxidant Defense and Autoimmunity Modulation:

Selenium plays a vital role in immune regulation by supporting the activity of selenoproteins, especially glutathione peroxidases and thioredoxin reductases, which protect immune cells from oxidative damage. These enzymes help neutralize reactive oxygen species (ROS) generated during inflammation, preserving immune cell integrity and function.

In the context of autoimmunity, selenium has shown promising immunomodulatory effects. Clinical studies, particularly in patients with Hashimoto’s thyroiditis, have demonstrated that selenium supplementation can reduce thyroid-specific autoantibodies and enhance regulatory T cell (Treg) function, indicating a potential role in restoring immune tolerance [47]. Selenium also influences the proliferation and differentiation of T cells and enhances the cytotoxic activity of natural killer (NK) cells, further reinforcing its protective role in host defense and immune homeostasis.

#### 2.2.3 Iron in Immune Cell Proliferation and Pathogen Defense:

Iron is a critical micronutrient for immune function, playing a central role in the proliferation and maturation of immune cells, particularly lymphocytes. It is essential for the synthesis of DNA and for the energy metabolism required by rapidly dividing immune cells. Iron also supports the microbial killing activity of phagocytes, such as macrophages and neutrophils, by enabling the generation of reactive oxygen species (ROS) through the Fenton reaction [48].

Iron deficiency can impair the function of innate and adaptive immune cells. Neutrophils may exhibit reduced chemotaxis and bactericidal activity, while T cell responses can become blunted due to impaired proliferation. This immunosuppressed state increases susceptibility to infections, particularly in vulnerable populations such as children and pregnant individuals [49].

However, iron overload poses its own risks. Excess iron can foster the growth of certain pathogens and increase oxidative stress, thereby contributing to tissue damage and inflammation [50]. Maintaining iron homeostasis is therefore crucial—both deficiency and overload disrupt immune balance and increase the risk of infection or chronic inflammation.

#### 2.2.4 Copper in Antibody Production and Antimicrobial Effects:

Copper plays a multifaceted role in immune defense by supporting the enzymatic activity of several critical proteins involved in immune responses. It acts as a cofactor for enzymes such as superoxide dismutase (SOD), which helps neutralize reactive oxygen species and reduces oxidative stress during immune activation. Copper also contributes to mitochondrial respiration and energy production, which are essential for the proliferation and function of immune cells [34].

Copper is particularly important for neutrophil and macrophage activity. These innate immune cells rely on copper-dependent enzymes to generate reactive oxygen intermediates needed for the destruction of pathogens. Additionally, copper is involved in the maturation and function of B cells and in the production of antibodies, highlighting its influence on humoral immunity.

Copper deficiency has been associated with impaired immune responses, increased susceptibility to infections, and decreased white blood cell counts. On the other hand, excessive copper levels can be cytotoxic and disrupt immune homeostasis, reinforcing the need for tightly regulated copper levels within the body.

#### 2.2.5 Magnesium in Inflammation Regulation and Immune Homeostasis:

Magnesium plays a vital role in immune regulation through its involvement in over 300 enzymatic processes. It contributes to both innate and adaptive immune responses by regulating the activity of immune cells, including lymphocytes and macrophages. Magnesium is also essential for maintaining the stability of cell membranes and modulating inflammatory signaling pathways. A deficiency can impair immune cell activation and cytokine regulation, leading to chronic low-grade inflammation and an increased risk of immune dysfunction [51].

### 2.3 Micronutrients in Viral Immunity and Aging

Micronutrients also play crucial roles in the body’s defense against viral infections. Key nutrients like vitamins A, C, D, E, as well as zinc and selenium, help maintain epithelial barriers, enhance antibody responses, and regulate inflammatory processes during viral challenges. Deficiencies in these micronutrients correlate with worse outcomes in viral infections such as influenza and SARS-CoV-2 [52]. In aging populations, where immune function naturally declines, multivitamin and mineral supplementation has been shown to improve immune markers, including increased plasma zinc and reduced inflammation [4].

### 2.4 Micronutrients as Direct Immune Modulators

Recent evidence suggests that micronutrients act not only as cofactors but also as direct immune signals. Nutrients have been conceptualized as “Signal 4” in T cell immunity, influencing T cell metabolism, differentiation, and memory formation [53]. This emerging framework highlights the significant role of nutritional status in shaping the quality, strength, and longevity of adaptive immune responses.

Overall, micronutrients are integral to the proper functioning of the immune system, influencing barrier integrity, immune cell development, inflammatory regulation, and pathogen defense. Deficiencies in vitamins A, C, D, E, B complex, and trace elements like zinc, selenium, iron, copper, and magnesium can impair immune responses and increase susceptibility to infections and autoimmune disorders. Continued research is needed to understand the synergistic effects of micronutrients and the potential of targeted supplementation to optimize immune health, particularly in vulnerable populations such as the elderly and individuals with chronic illnesses.

## 3. Macronutrients and Immune Regulation

Macronutrients—proteins, fats, and carbohydrates—are fundamental not only as energy sources but also as direct modulators of immune function. They influence immune cell metabolism, inflammatory pathways, and interact with the gut microbiota, all of which are critical for maintaining immune homeostasis.

### 3.1 Proteins and Amino Acids: Building Blocks of Immunity

Proteins and their constituent amino acids are essential not only as structural components but also as functional regulators of the immune system. They provide the building blocks for immune cell membranes, receptors, signaling molecules, and antibodies. Specific amino acids such as arginine, glutamine, and tryptophan have immunomodulatory roles that extend beyond basic nutrition. Arginine is critical for T cell proliferation and is a precursor for nitric oxide, which aids in macrophage-mediated pathogen destruction. Glutamine serves as a major energy source for rapidly dividing immune cells, including lymphocytes and intestinal epithelial cells, while tryptophan metabolism influences immune tolerance and inflammatory tone through the kynurenine pathway [54].

Disruptions in amino acid availability or metabolism, whether due to malnutrition, chronic disease, or metabolic imbalance, can impair immune cell activation and cytokine signaling, potentially shifting the immune environment toward a pro-inflammatory state. As a result, amino acid metabolism is increasingly being explored as a therapeutic target to restore immune balance, particularly in chronic inflammatory and cancer settings [55]. This highlights the importance of adequate protein intake and balanced amino acid profiles for sustaining effective immune defense and regulation.

### 3.2 Fats: Pro- and Anti-Inflammatory Effects

Dietary fats play a multifaceted role in immune regulation, with different types of fats exerting opposing effects on inflammatory pathways. Omega-3 polyunsaturated fatty acids (PUFAs), particularly eicosatetraenoic acid (EPA) and docosahexaenoic acid (DHA), are known for their anti-inflammatory properties. These fatty acids are incorporated into immune cell membranes, where they influence membrane fluidity and modulate the production of lipid mediators such as prostaglandins and leukotrienes. More importantly, they serve as precursors to specialized pro-resolving mediators (SPMs) like resolvins, protectins, and maresins, which actively promote the resolution of inflammation rather than merely suppressing it [55]. Omega-3s also strengthen gut barrier integrity and promote a favorable gut microbial composition, supporting immune tolerance and reducing systemic inflammation.

Conversely, saturated fatty acids (SFAs), commonly found in animal products and processed foods, have been associated with the activation of innate immune receptors such as Toll-like receptor 4 (TLR4). This receptor activation leads to downstream signaling cascades involving NF-κB and the production of pro-inflammatory cytokines like TNF-α and IL-6, which can contribute to chronic low-grade inflammation [8, 21, 56]. While early studies suggested a direct interaction between SFAs and TLR4, emerging evidence indicates that the inflammatory response may instead result from altered lipid metabolism or secondary signaling pathways, illustrating the complexity of how dietary fats influence immune function [19,21,42,56]. These contrasting effects highlight the importance of fat quality in the diet, where the balance between pro-inflammatory and anti-inflammatory fatty acids may shape immune outcomes and chronic disease risk.

### 3.3 Carbohydrates: Energy Source and Immune Modulator

While carbohydrates serve as essential fuel for immune cells, their quality and composition significantly impact immune regulation. Glucose availability can directly influence immune regulation by promoting regulatory T cell (Treg) differentiation, which supports intestinal homeostasis and immune tolerance [57]. Dietary fiber, a complex carbohydrate, is fermented by gut microbiota into short-chain fatty acids (SCFAs) such as butyrate, propionate, and acetate. These SCFAs modulate immune responses by enhancing gut epithelial integrity, increasing regulatory T-cell numbers, and decreasing the expression of inflammatory cytokines, thereby maintaining mucosal homeostasis [15,21,58].

Conversely, diets low in fiber and high in refined carbohydrates are associated with reduced SCFA production, leading to compromised gut barrier function and increased susceptibility to inflammation. The broader impact of carbohydrates on gut-immune interactions and metabolic health will be discussed in subsequent sections.

In summary, macronutrients actively participate in immune regulation beyond their caloric contributions. Proteins and amino acids are fundamental for immune cell growth and function; omega-3 fatty acids reduce inflammation and support gut immunity; saturated fats can induce pro-inflammatory pathways; and dietary fiber influences immune responses through SCFA production. The interplay between macronutrient intake, gut microbiota composition, and immune function underscores the importance of balanced nutrition for maintaining immune health—a complex interaction that will be explored further in the following sections.

## 4. Gut Microbiota and Immune Function

The gut microbiota serves as a critical regulator of immune function, influencing the development, maturation, and modulation of immune responses. Interactions between dietary components, microbial metabolites, and host immunity play a central role in maintaining immune homeostasis or, conversely, driving immune dysregulation when imbalanced.

### 4.1 Dietary Fiber, Short-Chain Fatty Acids (SCFAs), and Immune Modulation

Dietary fibers are fermented by gut bacteria to produce short-chain fatty acids (SCFAs)—primarily butyrate, acetate, and propionate—which serve as key immunomodulatory metabolites [58]. SCFAs promote regulatory T cell (Treg) differentiation, suppress pro-inflammatory cytokine production, and enhance gut epithelial integrity, helping to maintain mucosal immune tolerance.

Butyrate, in particular, strengthens the gut barrier and reduces systemic inflammation by influencing immune cell gene expression and suppressing Th17 cell activity. SCFAs also regulate T cell metabolism, shifting it from glycolysis to oxidative phosphorylation, favoring anti-inflammatory pathways [59].

### 4.2 Diet-Microbiota Interactions and Immune Dysregulation

Diet is one of the most significant factors shaping the gut microbiota. High-fiber, plant-based diets increase microbial diversity and SCFA production, promoting anti-inflammatory effects and barrier integrity [8–10]. In contrast, Western diets—rich in saturated fats and refined carbohydrates—reduce microbial diversity, promote the expansion of pro-inflammatory bacterial strains, and increase gut permeability, contributing to “leaky gut” and systemic inflammation. Such dysbiosis has been implicated in various immune-related conditions, including obesity, diabetes, and autoimmune diseases. The chronic low-grade inflammation triggered by gut barrier dysfunction and microbial imbalance underscores the importance of diet in immune regulation [8,13].

### 4.3 Microbiota, Epigenetics, and Autoimmune Disease Risk

Emerging research reveals that gut microbiota also influence the immune system through epigenetic mechanisms. SCFAs, particularly butyrate, act as histone deacetylase (HDAC) inhibitors, affecting gene expression in immune cells and promoting regulatory pathways [59,60]. Furthermore, microbial metabolites may modulate DNA methylation patterns, impacting immune cell development and autoimmune risk [35–37]. These epigenetic modifications provide a potential mechanistic link between diet, microbiota, and long-term immune health.

### 4.4 Nutrient-Microbiota-Immune Axis: A Dynamic System

Macronutrients also shape the gut-immune axis. High intake of animal protein and saturated fats favors pro-inflammatory bacterial species, while fiber and polyphenols support beneficial microbes and SCFA production [13]. These shifts influence immune activation and inflammation, reinforcing the importance of dietary patterns in maintaining gut and systemic immune health.

In summary, the gut microbiota is a central mediator of immune regulation, heavily influenced by dietary intake. Through the production of SCFAs and epigenetic modulation, gut microbes shape immune responses, protect against inflammation, and maintain mucosal tolerance. Dysbiosis and dietary imbalances disrupt this relationship, contributing to immune dysregulation and chronic diseases. The following section will further explore how epigenetic mechanisms, influenced by both diet and the gut microbiota, regulate immune function and disease susceptibility.

## 5. Epigenetics and Nutritional Immunology

**Figure 2:**
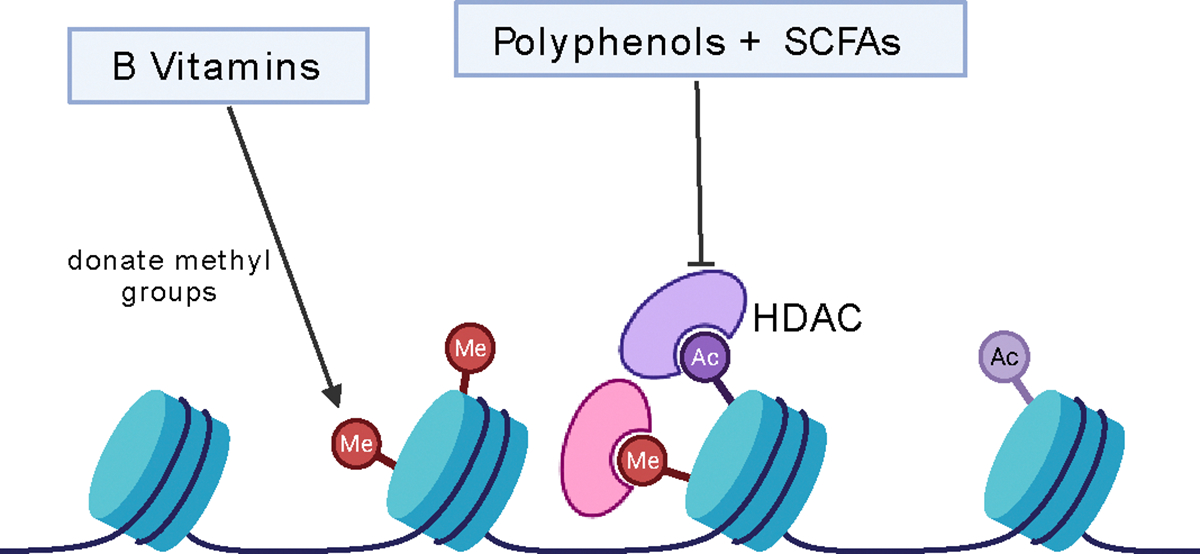
Epigenetic Regulation of Immunity by Dietary Nutrients: This figure illustrates how specific dietary components influence immune function through epigenetic mechanisms. B vitamins (B6, B12, folate) serve as methyl donors, supporting DNA methylation. Polyphenols (from berries, green tea) and SCFAs like butyrate act as histone deacetylase (HDAC) inhibitors, enhancing histone acetylation and promoting anti-inflammatory gene expression. Together, these epigenetic modifications regulate immune cell differentiation, cytokine production, and immune tolerance.

Epigenetics—heritable changes in gene expression without altering the DNA sequence—plays a crucial role in regulating immune responses. Key epigenetic mechanisms include DNA methylation, histone modifications, and microRNA (miRNA) regulation, all of which influence immune cell development, cytokine production, and inflammation [5–7,61–65]. Nutrition is increasingly recognized as a powerful modulator of these epigenetic processes, shaping immune function and disease susceptibility (Figure 2).

### 5.1 DNA Methylation and Immune Cell Regulation

DNA methylation, the addition of methyl groups to cytosine residues in DNA, is a key epigenetic mechanism essential for immune cell development, lineage specification, and functional stability. Methylation patterns guide the differentiation of hematopoietic stem cells into various immune cell types, including T cells, B cells, macrophages, and dendritic cells. For example, proper methylation of the FOXP3 gene is critical for the development and function of regulatory T cells (Tregs), which play a central role in maintaining immune tolerance and preventing autoimmunity [66].

Aberrant or dysregulated methylation patterns—either hypermethylation or hypomethylation—can lead to inappropriate gene silencing or activation, contributing to immune dysfunction. Such epigenetic alterations have been linked to autoimmune diseases like systemic lupus erythematosus (SLE) and multiple sclerosis, where the immune system fails to distinguish self from non-self [7,35].

Nutrients involved in one-carbon metabolism, including folate, vitamins B6 and B12, and choline, act as methyl donors or cofactors in the methylation cycle. Adequate intake of these micronutrients ensures sufficient S-adenosylmethionine (SAM), the universal methyl donor for DNA methyltransferases (DNMTs). A deficiency in these nutrients reduces methyl availability, potentially altering immune-related gene expression and impairing tolerance mechanisms [61]. This illustrates how nutritional status can have long-term impacts on immune function through epigenetic regulation.

### 5.2 Histone Modifications and Inflammatory Gene Regulation

Histone modifications—including acetylation, methylation, phosphorylation, and ubiquitination—regulate the accessibility of DNA wrapped around histone proteins, thereby influencing gene transcription. Acetylation of histone tails by histone acetyltransferases (HATs) generally relaxes chromatin and promotes gene expression, while deacetylation by histone deacetylases (HDACs) condenses chromatin, repressing transcription. These dynamic modifications are crucial in innate immunity, particularly in determining how antigen-presenting cells such as macrophages and dendritic cells respond to stimuli [62].

Diet and microbial metabolites can influence these histone marks. For instance, short-chain fatty acids (SCFAs), especially butyrate, produced by gut microbiota during fiber fermentation, act as HDAC inhibitors. By inhibiting HDACs, butyrate enhances histone acetylation and promotes the expression of anti-inflammatory genes in immune cells. This mechanism provides a molecular explanation for how diet—particularly high-fiber, plant-based, or Mediterranean-style diets—can modulate immune responses at the epigenetic level.

Moreover, polyphenols found in foods such as berries, tea, and extra virgin olive oil have been shown to influence histone modification patterns, reducing the expression of pro-inflammatory genes and influencing immune cell polarization. Together, these findings establish a compelling link between dietary intake, microbiota-derived metabolites, and epigenetic regulation of immune function [62].

### 5.3 MicroRNAs (miRNAs), Diet, and Immune Modulation

MicroRNAs (miRNAs) are small, non-coding RNA molecules that regulate gene expression post-transcriptionally by binding to target messenger RNAs (mRNAs) and either degrading them or blocking their translation. These regulatory molecules are essential for fine-tuning immune responses, influencing key processes such as T cell differentiation, B cell maturation, cytokine production, and innate immune signaling [63].

MiRNAs help maintain immune homeostasis by modulating inflammatory pathways. For example, pro-inflammatory miRNAs like miR-155 promote Th1 and Th17 responses, while anti-inflammatory miRNAs like miR-146a and miR-21 act as negative regulators of Toll-like receptor (TLR) and NF-κB signaling pathways, helping to prevent excessive inflammation [63].

Emerging evidence demonstrates that dietary components can significantly alter miRNA expression profiles. This regulation occurs through various bioactive compounds such as polyphenols, omega-3 fatty acids, and vitamins. Polyphenols from berries and green tea, for instance, can downregulate miR-155 and upregulate anti-inflammatory miRNAs like miR-146a, contributing to a shift toward immune tolerance and reduced inflammatory tone. Similarly, omega-3 fatty acids influence miRNA expression in T cells and macrophages, promoting anti-inflammatory gene expression profiles [63,64].

This field of research, known as nutrimiromics, explores how diet-derived molecules regulate immune gene networks via miRNA pathways. It underscores the potential of dietary interventions not only to reduce inflammation but also to modulate disease outcomes in chronic immune-related conditions. As the field advances, miRNA signatures may even serve as biomarkers for nutritional status and immune health, paving the way for more personalized dietary strategies to support immune balance [64].

### 5.4 Dietary Patterns, Epigenetics, and Immune Health

Healthy dietary patterns, such as the Southern European Atlantic Diet—rich in fish, fiber, polyphenols, and micronutrients—promote beneficial epigenetic changes linked to reduced inflammation and improved immune regulation [65]. In contrast, diets high in processed foods, saturated fats, and refined sugars may disrupt epigenetic marks, contributing to immune dysregulation.

Additionally, emerging evidence suggests that targeted nutritional interventions may modify epigenetic aging (epigenetic clocks), potentially delaying immune senescence and reducing the risk of chronic inflammatory diseases [67].

Overall, nutrition and dietary patterns profoundly influence epigenetic mechanisms that regulate immune responses. Through DNA methylation, histone modifications, and miRNA expression, diet modulates immune cell function, inflammatory pathways, and disease risk. This growing field of nutritional epigenetics offers promising avenues for preventive strategies and therapeutic interventions in immune-related and metabolic diseases—topics explored in the following section.

## 6. Dietary Patterns and their Impact on Immunity and Metabolic Health

**Figure 3:**
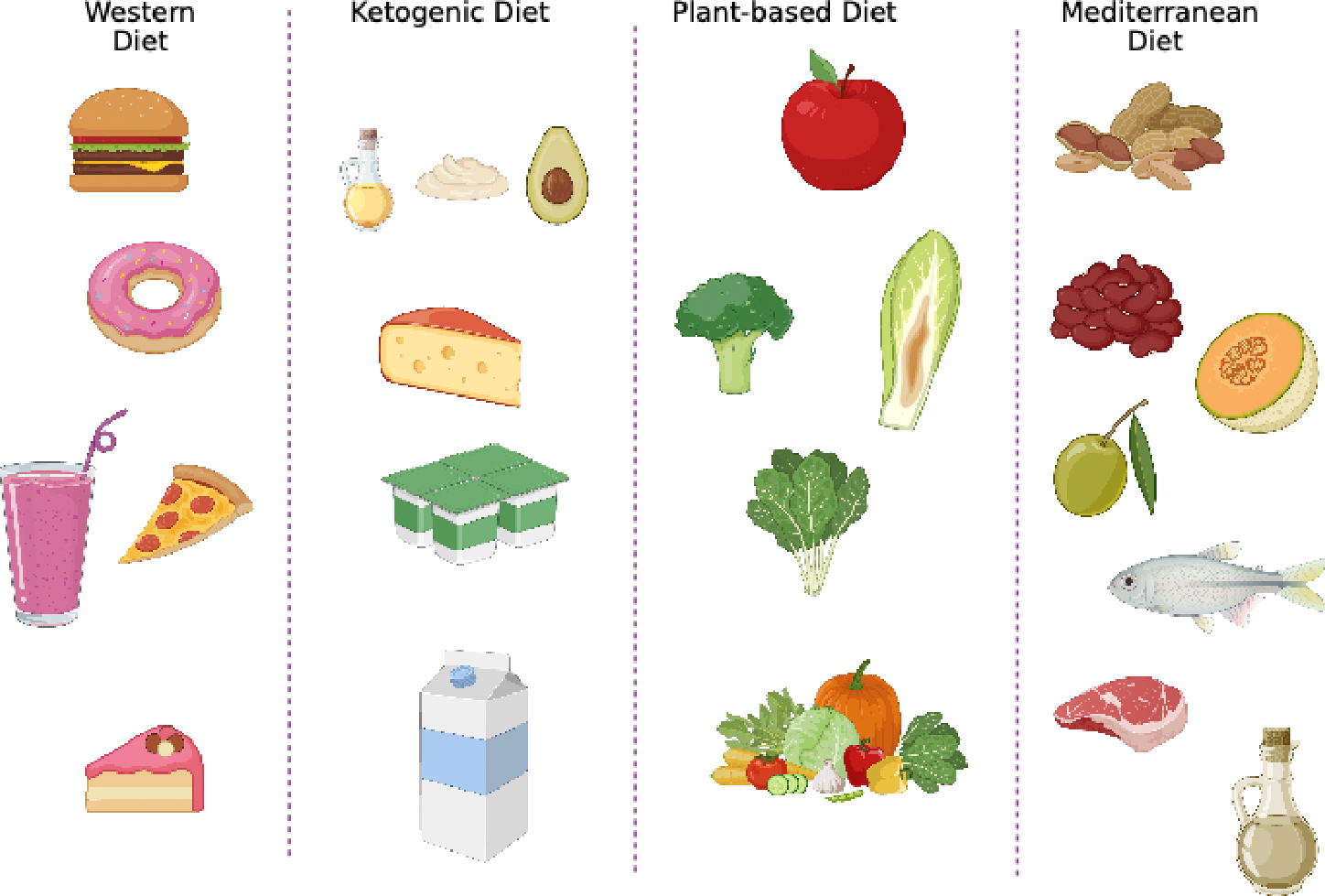
Comparison of Western, Ketogenic, Mediterranean, and Plant-Based Diets on Immune Health: This figure compares four dietary patterns in relation to their effects on inflammation, gut microbiota, and immune function. The Western diet is associated with increased inflammation and microbial dysbiosis. The Mediterranean and plant-based diets promote microbial diversity, enhance short-chain fatty acid production, and reduce systemic inflammation. Ketogenic diets demonstrate emerging immunometabolic effects but may negatively affect gut microbial richness if fiber is insufficient.

Beyond individual nutrients, overall dietary patterns profoundly influence immune responses, gut microbiota composition, systemic inflammation, and long-term metabolic health. Rather than acting in isolation, nutrients are consumed as part of broader dietary frameworks that modulate the gut-immune-metabolic axis. Some patterns enhance immune resilience and prevent disease, while others promote chronic inflammation and immune dysfunction (Figure 3).

### 6.1 Western Diet: Pro-Inflammatory and Immune Disruption

The Western diet, prevalent in industrialized nations, is characterized by high consumption of saturated fats, refined carbohydrates, added sugars, and ultra-processed foods, coupled with low intake of fiber, vegetables, and micronutrients. This dietary pattern has been strongly associated with gut dysbiosis, a decrease in microbial diversity, and impaired production of beneficial metabolites such as SCFAs [68].

Western diets contribute to increased intestinal permeability (“leaky gut”) and systemic translocation of bacterial endotoxins like lipopolysaccharide (LPS), which activate immune pathways and lead to low-grade, chronic inflammation. This inflammatory state disrupts immune tolerance and promotes the development of metabolic disorders, including obesity, insulin resistance, type 2 diabetes, and non-alcoholic fatty liver disease (NAFLD) [69].

Moreover, the Western diet reprograms macrophage metabolism and promotes Th17 polarization, exacerbating autoimmune susceptibility. Emerging research suggests that even short-term consumption of a Western diet can impair immune memory formation and reduce vaccine efficacy, further illustrating its detrimental impact on immune competence.

### 6.2 Mediterranean Diet: Anti-Inflammatory and Immune Protective

The Mediterranean diet, traditionally followed in Southern Europe, emphasizes the consumption of fruits, vegetables, whole grains, legumes, nuts, olive oil, and fish, while limiting red meats and refined sugars. This pattern is consistently associated with reduced inflammation, improved cardiovascular health, and lower incidence of metabolic and autoimmune diseases.

From an immunological perspective, the Mediterranean diet enhances gut microbial diversity and supports the growth of SCFA-producing bacteria such as *Faecalibacterium prausnitzii* and *Bifidobacteria* [70,71]. SCFAs derived from fiber fermentation exert immunomodulatory effects, including Treg induction and suppression of inflammatory cytokines like IL-6 and TNF-α. Studies by Nagpal, et al. [70] and Merra, et al. [71] confirm that adherence to a Mediterranean dietary pattern positively modulates gut microbiome composition, improving immune function and reducing oxidative stress.

Additionally, the diet is rich in polyphenols and omega-3 fatty acids, which modulate immune cell signaling, suppress NF-κB activation, and maintain epithelial barrier integrity. These mechanisms collectively contribute to reduced risk of chronic inflammation and immune-related conditions.

### 6.3. Plant-Based Diets: Enhancing Microbial Diversity and Reducing Inflammation

Plant-based diets, which include vegetarian and vegan patterns, emphasize the consumption of whole plant foods while excluding or minimizing animal products. These diets are naturally high in fiber, antioxidants, and phytonutrients, and low in saturated fat, making them favorable for immune and metabolic health.

Research demonstrates that plant-based diets increase the abundance of beneficial gut microbes, such as *Akkermansia* and *Lactobacillus*, which contribute to SCFA production and gut barrier function [72]. Sidhu, et al. [72] showed that plant-based diets enhance microbial richness and decrease pro-inflammatory markers, supporting their role in modulating immune responses [72].

In addition to enhancing microbial ecology, plant-based diets have been associated with reduced biomarkers of inflammation, such as C-reactive protein (CRP) and interleukin-6 (IL-6), and improved glycemic control. These benefits may reduce the risk and severity of autoimmune, inflammatory, and metabolic diseases.

### 6.4 Low-Carbohydrate and Ketogenic Diets: Emerging Immunometabolic Effects

Low-carbohydrate and ketogenic diets—marked by very low carbohydrate intake and high fat consumption—are gaining interest for their effects on metabolic and immune function. These diets induce a metabolic shift from glucose to ketone bodies (e.g., β-hydroxybutyrate), which have been shown to possess anti-inflammatory properties.

Ketogenic diets may enhance CD8+ T cell function and memory by promoting oxidative phosphorylation over glycolysis, a process termed immunometabolic reprogramming [73]. This shift may improve immune resilience and reduce chronic inflammation, although findings are still emerging and context dependent.

While some data support the immunomodulatory potential of ketogenic diets, concerns remain regarding their long-term impact on gut microbiota diversity, as they are often low in fermentable fiber. Therefore, further research is needed to balance metabolic benefits with immune and microbiome outcomes.

### 6.5 Dietary Patterns and Autoimmune/Inflammatory Conditions: IBD as an Example

Autoimmune and inflammatory diseases, such as inflammatory bowel disease (IBD), are heavily influenced by dietary patterns through their impact on the gut-immune axis. In IBD, the Western diet exacerbates symptoms by fostering dysbiosis, reducing microbial resilience, and promoting mucosal inflammation [74].

Conversely, anti-inflammatory dietary patterns such as the Mediterranean and plant-based diets may help restore microbial diversity and promote immune tolerance. These diets are associated with reductions in intestinal inflammation, enhanced epithelial barrier function, and increased SCFA production, which collectively support remission and symptom control in IBD patients.

By altering gut microbiota composition, epigenetic marks, and inflammatory signaling pathways, dietary patterns offer a modifiable tool in managing chronic immune-mediated diseases. These findings underscore the potential of personalized nutrition as a therapeutic strategy in immune health.

In summary, dietary patterns exert profound effects on the immune system, primarily through modulating the gut microbiota, metabolic pathways, and inflammatory responses. Diets rich in fiber, healthy fats, and phytonutrients—such as the Mediterranean and plant-based diets—support immune health, while Western dietary patterns promote chronic inflammation and immune dysfunction. These interactions underscore the importance of diet quality in preventing immune-related and metabolic diseases.

## 7. Nutrition, Immune Dysregulation, and Metabolic Disorders

Metabolic disorders such as obesity and type 2 diabetes are strongly associated with chronic immune dysregulation, much of which is driven by poor dietary quality, excessive energy intake, and resulting alterations in gut microbiota and immune signaling. These disorders exemplify the interplay between metabolism and immunity—often referred to as *immunometabolism*—where overnutrition and systemic inflammation exacerbate each other in a self-perpetuating cycle. Understanding how nutritional factors impact this relationship is critical for developing preventive and therapeutic strategies.

### 7.1 Adipose Tissue Inflammation and Insulin Resistance

Obesity is marked by hypertrophic expansion of adipose tissue, which is not metabolically inert but actively participates in immune responses. As adipocytes enlarge, they release chemokines that attract immune cells—particularly M1-polarized macrophages and pro-inflammatory T cells. These immune cells produce cytokines such as TNF-α and IL-6, which disrupt insulin signaling pathways and impair glucose uptake [74,75]. This process, termed *meta-inflammation*, represents a chronic, low-grade inflammatory state that underlies insulin resistance and increases the risk for type 2 diabetes [15,21]. Furthermore, this inflammation can extend beyond adipose tissue to affect vascular endothelium, liver, and muscle, amplifying metabolic dysfunction.

### 7.2 Gut Dysbiosis, Endotoxemia, and Liver Inflammation

The composition and function of the gut microbiota are highly sensitive to dietary inputs. Diets rich in saturated fats and low in fiber—typical of the Western dietary pattern—disrupt microbial balance, decreasing beneficial bacteria and promoting growth of pro-inflammatory species [76]. This dysbiosis increases intestinal permeability, a condition often described as “leaky gut,” which allows microbial products like LPS to enter the bloodstream. The resulting metabolic endotoxemia activates systemic inflammation and is a known driver of insulin resistance and liver injury. In the liver, this contributes to progression from simple steatosis to inflammatory conditions such as non-alcoholic steatohepatitis (NASH), a key comorbidity of obesity.

### 7.3 Hyperglycemia, Oxidative Stress, and Immune Dysfunction

In type 2 diabetes, chronic hyperglycemia creates an oxidative environment that impairs immune responses. Elevated glucose levels promote the generation of reactive oxygen species (ROS), which damage cellular proteins, lipids, and DNA [77]. This oxidative stress compromises the function of neutrophils, macrophages, and lymphocytes, reducing host defense capabilities. Moreover, it triggers the activation of stress-sensitive pathways (e.g., NF-κB), which amplify inflammation. The sustained inflammatory milieu contributes to diabetic complications such as neuropathy, retinopathy, nephropathy, and cardiovascular disease, linking metabolic and immune dysfunction at the molecular level [77–79].

### 7.4 Nutrient Sensing Pathways and Chronic Inflammation

Nutrient-sensing pathways such as mTOR (mechanistic target of rapamycin), AMPK (AMP-activated protein kinase), and NF-κB (nuclear factor kappa B) translate metabolic signals into immune actions. Chronic overnutrition activates mTOR and NF-κB, skewing immune responses toward pro-inflammatory phenotypes (e.g., Th1 and Th17 cells) and promoting macrophage polarization toward the M1 inflammatory state [80]. Meanwhile, suppressed AMPK signaling reduces anti-inflammatory processes. Together, these shifts contribute to chronic immune activation and low-grade inflammation, characteristic of metabolic syndrome. These nutrient-sensing pathways thus serve as molecular links between diet, energy metabolism, and immune homeostasis.

### 7.5 Autoimmune Diseases and Nutritional Immune Dysregulation

In addition to metabolic disorders, autoimmune diseases represent another consequence of immune dysregulation influenced by diet, gut microbiota, and epigenetics. Altered gut microbiota and increased intestinal permeability may trigger autoimmune responses by exposing the immune system to self-antigens. Nutrient deficiencies, particularly in vitamin D and selenium, have been linked to increased risk of autoimmune conditions such as multiple sclerosis, rheumatoid arthritis, and lupus [4]. Furthermore, epigenetic modifications influenced by diet and microbial metabolites may alter immune cell function, contributing to autoimmunity.

Together, these findings underscore the complex interplay between nutrition, immune function, and metabolic health. Poor dietary patterns drive chronic inflammation, gut dysbiosis, and oxidative stress, leading to both metabolic diseases and autoimmune conditions. Understanding these mechanisms is critical for developing nutritional strategies to prevent and manage immune-related chronic diseases—a concept explored in the concluding section.

## 8. Future Directions

This review emphasizes the critical role of nutrition, gut microbiota, and epigenetic regulation in shaping immune function and maintaining metabolic health. Micronutrients and macronutrients are essential for immune cell development, inflammation regulation, and maintaining physical barriers against pathogens. Dietary patterns significantly impact immune resilience, with fiber-rich and nutrient-dense diets supporting immunity, while Western diets promote dysbiosis and chronic inflammation. Despite growing evidence, there are notable gaps in understanding the long-term effects of specific dietary patterns on immune system regulation, particularly regarding epigenetic modifications, which are often studied only acutely. Future research should aim to identify epigenetic markers that predict individual immune responses to different diets and investigate how modulating gut microbiota through personalized nutrition can reduce inflammation, improve insulin sensitivity, and prevent autoimmune diseases. Addressing these gaps may pave the way for targeted dietary interventions that promote immune health and prevent chronic diseases such as obesity and diabetes.

## Key Points

- Micronutrients like vitamins A, C, D, E, B-complex, zinc, selenium, and iron are crucial for immune cell function, antioxidant defense, and inflammation control.
- Macronutrients influence immunity — protein supports immune cell growth, omega-3s are anti-inflammatory, saturated fats promote inflammation, and fiber enhances gut health.
- Gut microbiota regulate immunity by producing short-chain fatty acids (SCFAs), promoting regulatory T cells, and balancing inflammatory responses.
- High-fiber, plant-based, and Mediterranean diets support immune resilience, while Western diets drive chronic inflammation and immune dysfunction.
- Epigenetic mechanisms, including DNA methylation, histone modification, and miRNA regulation, link nutrition to long-term immune modulation and disease risk.
- Obesity and diabetes result from immune dysregulation, chronic inflammation, gut microbiota alterations, and oxidative stress driven by poor diet quality.
- Future nutritional strategies and personalized diets targeting the microbiome and epigenetic pathways may reduce inflammation and prevent immune-related chronic diseases.
